# Brain-derived neurotrophic factor and C-reactive protein (CRP) biomarkers in suicide attempter and non-attempter major depression disorder (MDD) patients

**DOI:** 10.1186/s12991-024-00511-3

**Published:** 2024-07-22

**Authors:** Seyed Hassan Saadat, Mohammad Javanbakht, Shima Shahyad

**Affiliations:** 1 Nephrology and Urology Research Center, Clinical Science Institute, Baqiyatallah University of Medical Sciences, Tehran, Iran; 2 Neuroscience Research Center, Baqiyatallah University of Medical Sciences, Tehran, Iran

**Keywords:** Brain-derived neurotrophic factor, C-reactive protein, Major depressive disorder, Suicide

## Abstract

**Background:**

In the available literature, levels of BDNF and CRP have been reported to correlate with suicide in depressive patients but there are inconsistencies in the results. We aimed to evaluate and compare BDNF and CRP concentrations in MDD patients with(MDD + SA) and without suicide attempts (MDD-SA) and healthy controls.

**Methods:**

30 (MDD + SA) patients, 30 (MDD-SA) patients, and 26 healthy controls were enrolled in the study. Age, sex, and BMI of patients were recorded. Blood sample was obtained for measurement of BDNF and CRP. Smoking and drug history, family history of suicide, and history of self-harm were also documented. Data were analyzed with SPSS version 22 and R version 4.1.1.

**Results:**

86 patients in three groups were evaluated (mean age: 28.45 ± 9.27 years, 56.71% female). Baseline and demographic parameters except for self-harm (40%, 3.3%, and 0% for MDD + SA, MDD-SA, and healthy controls, respectively, p = 0.001) did not differ between groups. CRP level was not significantly different between groups. BDNF showed a significant difference between groups (17.35, 16.45, and 19.43 for three groups, respectively, p < 0.001). An increase in BDNF decreased the odds of both depression and suicide. Roc curve showed excellent power for BDNF in discriminating MDD groups With healthy group.Roc can notdicrimiate MDD + SA and MDD-SA.

**Conclusion:**

In our study, the concentration of BDNF differed significantly between depressed patients with/without suicide attempts and healthy controls which shows the association of BDNF with depression development and not suicide attempts. We could not find any association between CRP level and suicide attempt but still larger cohorts are needed for a definite conclusion.

## Background

Suicide is globally known as a major public health concern with approximately 800,000suicide-related deaths annually [1]. Several recent research studies on suicidal behavior have reported that suicide is a major or one of the leading causes of mortality at all ages, nationally or worldwide [2]. A national study in Iran has shown that disability-adjusted life years (DALY) for suicidal and self-harm behaviors are 206.2 per 100,000 populations [3].

The prevalence of suicide in patients with psychiatric disorders is about ten-fold higher than the general population. 60–98% of suicide attempts accompany psychiatric disorders mainly depression [4]. Depression and suicide lead to a loss of function in daily activities, cause psychological distress, and accompany substance abuse [5]. demographic and clinical data provide valuable information in predicting suicide, but it is beneficial to support it with biological markers and provide stronger data. This has triggered the search for more valid and reliable biological markers that could predict suicidal behavior and possible prevention [6].

Brain-derived neurotrophic factor (BDNF) is an agent thatregulates connections of nervous pathways and cells in the brain [7]. Reports have claimed that BDNF is remarkably low in patients who havecommitted suicide. Postmortem studies on suicide victims’ brains have also revealed supporting evidence for the role of BDNF in suicide [8]. It is speculated that the expression and function of BDNF is decreased in patients with suicide attempts. Still, there is no consensus regarding the role of BDNF in suicide and questions still exist on this issue.

Increasing reports are being published on the role of inflammatory processes in the development of depression and suicide. Clinical observations and epidemiologic investigations [9–11]. Have shown that innate immune response is probably involved in the development of suicidal behavior. C-reactive protein (CRP) as a marker of systemic inflammation has been investigated in suicide studies. Observations on the role of CRP in suicide have yielded conflicting results. For instance, Vargas et al. [12] have shown no significant difference in CRP between patients with and without suicide history while on the other hand, O’Donovan et al. [13] have reported that depressed patients with high suicidal thoughts exhibit significantly higher levels of CRP compared to depressed patients with low suicidal thoughts or healthy controls, regardless of depression severity. They reported no significant difference in terms of CRP between non-suicidal depressed patients and healthy controls. Thus, more studies are needed to clarify the relationship of CRP and suicidal behavior in different regions and on different ethnicities.

So, we aimed to evaluate the role of BDNF and CRP in suicidal behavior among Iranian patients with major depressive disorder.

## Methods and materials

### Settings and participants

This exploratory observational study was conducted between March 2019 and October 2021. Eligible participants for this project included individuals aged 18 to 65 years diagnosed with MDD and admitted to the psychiatric sections of two government hospitals, Loghman and Baqiattalah, located in Tehran, Iranthe.Sample size was calculated using the following formula:$n = \frac{{{z}_{{1} - {a/2}}^{2} \times {P} \left( {1 - P} \right)}}{{d}^{2}}$ (Z1-a/2 = 1.96, d = 0.1 and p = 0.7). Khan et al.’s study [14] revealed an average accuracy rate close to 70%. With this percentage, a 5% Type I error rate, and an absolute error of 10% incorporated into the sample size calculation, the estimated sample size was initially set at 80 cases. This number was adjusted to 86 cases, accounting for a 5% final volume loss. The sample was then divided among three groups in the following proportions: 30, 30, and 26.A structured clinical interview, based on DSM-V^1^ [15] and conducted by a board-certified psychiatrist, was employed to select patients with MDD + SA and MDD-SA. New patient medical records from the hospital’s suicide section were reviewed until the desired sample size was reached. All selected patients had no history of psychotherapy or electroshock therapy, and they had abstained from specific medications, including psychiatric medications, antibiotics, steroids, etc., for at least four weeks prior to the study. Patients were excluded if they had a prior diagnosis of anxiety, depressive or bipolar disorders, personality disorders or if they had comorbid somatic conditions such as cerebrovascular diseases, coronary artery diseases, hyperlipidemia, atherosclerosis, chronic inflammatory disorders, diabetes mellitus, endocrine and metabolic disturbances, infectious diseases, malignancies, were pregnant or had given birth within the previous 12 months, had a body mass index exceeding 30 kg/m2, a recent history of substance abuse within the last year, a prior history of psychotherapy, patients with a history of suicide but no suicidal attempt during the course of the last depressive episode or if they refused to provide written informed consent to participate in the study.Following the application of these inclusion and exclusion criteria, a total of 30 MDD + SA patients and 30 MDD-SA patients (26 by drug overdose, one by chemicals, one by insecticide, one by opium, and one by phosphate) were selected for this study. Additionally, 26 healthy controls, matched in terms of age and sex, were recruited. The control group was screened for depression using the Beck Depression Inventory (BDI) II through an interview conducted by a board-certified psychiatrist.All procedures involving human participants adhered to ethical standards set by the institutional and/or national research committee, in accordance with the 1964 Helsinki Declaration and its later amendments or equivalent ethical standards. The study received approval from the ethics committee affiliated with Baqiyatallah University of Medical Sciences (IR.BMSU.BAQ.REC.1399.005), and written informed consent was obtained from all participating patients.

### Assessment of variables

Demographic data were collected either from medical records or through questionnaires administered to the control groups. Body Mass Index (BMI) was calculated as weight divided by height (kg/m2). Information on smoking, substance use, family history of suicide, and self-harm history was documented using a researcher-designed questionnaire, with yes–no questions, such as “Have you ever self-harmed?” (Yes/No).For the assessment of biological markers, The blood draw on the first morning after hospitalization was performed with eight hours of fasting. Suicidal thoughts were not asked during the blood draw, but the patient’s mental state was evaluated during the daily checkup, and those who were not in a stable psychological condition were excluded from the study.. Blood was collected in a coagulation-free tube through venipuncture for the analysis of Brain-Derived Neurotrophic Factor (BDNF). Upon arrival, blood samples were also collected for C-Reactive Protein (CRP) analysis. Blood samples were kept at room temperature for 15 min to clot and then centrifugation at the speed of 3000 round per minute was performed for 10 min. The temperature was lowered to −20 °C for keeping serum frozen. Measurement of BDNF levels was performed with an ELISA kit ((ZellBio GmbH, Germany) according to the instructions of the manufacturer. Spectrophotometric assessment of optical density at wavelength of 450 nm ± 2 nm was performed which was proportional to BDNF levels. Evaluations revealed that inter-assay and intra-assay BDNF variations were below 11% and 10%, respectively.The least detectable dose of BDNFwas 0.05 ng/ml. For CRP measurement, Bionik diagnostic systems CRP-LIA kit was used. The least detection value of CRP was 0.1 mg/l with both inter-assay and intra-assay variations of below 10%.

### Statistical analysis

Categorical variables were presented as frequencies (percentages). To assess the normality of the distribution, continuous variables were described as either median (interquartile range, IQR) or mean (± standard deviation, SD) for non-normal and normal distributions, respectively.Initially, the null hypothesis was set to test whether there were any differences among MDD + SA patients, MDD-SA patients, and healthy subjects in terms of BDNF and hs-CRP levels, as well as other baseline characteristics. To test this hypothesis, we used one-way analysis of variance or Kruskal–Wallis tests (when normality assumptions were violated) to compare the means of continuous characteristics among these three independent groups. After identifying statistical significance, post hoc Bonferroni tests were employed for pairwise mean comparisons. Additionally, associations between categorical variables were examined using Chi-square tests.Next, to estimate the crude effect size (Odds Ratio with a 95% confidence interval) of each variable, particularly CRP and BDNF markers, on the occurrence of MMD with and without suicide, univariate multinomial logistic regression models were utilized. Finally, to identify the risk factors associated with MMD with and without suicide, a multiple logistic regression model was constructed. In the final model, variable selection was based on improvements in Akaike’s Information Criterion (AIC). The prognostic accuracy of the final model was evaluated using the area under the receiver operating characteristic curves (AUROC). A two-sided p-value less than 0.05 was considered statistically significant. Data analysis was conducted using R version 4.1.1 and SPSS software version 22 (IBM Corp., Armonk, NY, USA).

## Results

### Demographics and baseline characteristics

A total of 86 individuals participated in this research, and they were categorized into three groups: MD + SA (30 participants), MD-SA (30 participants), and a healthy control group (26 participants). The average age of the participants was 28.45 ± 9.27 years. In total, 49 subjects (56.97%) were female, while 37 subjects (43.03%) were male. The distribution of sex did not exhibit any significant differences among the three study groups (p = 0.869).Various factors including weight, BMI, educational level, occupational status, marital status, smoking history, history of former substance abuse, and family history of suicide were also evaluated, and no significant differences were observed among the study groups (p-values > 0.05), with the exception of self-harm history. Additionally, it’s worth noting that the suicide group had a statistically significant difference in height compared to the control group. For a comprehensive overview of the demographic and baseline parameters in the three study groups, please refer to Table 1**.**

**Table 1:** Comparison of CRP, BDNF markers and baseline characteristics among three groups of study

Variables		MD + SA n = 30(35%)			MD-SA n = 30(35%)			Healthy controls^b^ n = 26(30%)		P-value^c^
		Mean ± SD or Median (Q1- Q3) or n (%)	OR + (95%CI)	P-value^a ^	Mean ± SD or Median (Q1- Q3) or n (%)	OR + (95%CI)	P-value^a^	Mean ± SD or Median (Q1- Q3) or n (%)	OR^+^ (95%CI) (Reference)	
CRP (mg/L)		0.91(0.41–4.13)	1.04(0.98,1.10)	0.180	1.11(0.39–2.95)	1.03(0.97, 1.10)	0.270	0.87(0.37–1.65)	-	0.643
BDNF (ng/ml)		17.35 ± 2.43	0.68(0.53,0.87)	0.002	16.45 ± 3.15	0.60(0.46,0.78)	< 0.001	19.65 ± 2.02	-	< 0.001
Age (year)		29.03 ± 11.29	1.003(0.95,1.06)	0.904	27.33 ± 8.33	0.98(0.93,1.04)	0.565	28.73 ± 7.74	-	0.714
Sex (female)		16(53%)	0.84(0.29, 2.41)	0.743	18(60%)	1.10(0.38,3.20)	0.861	15(58%)	-	0.869
Height(cm)		173 ± 10	1.07(1.01,1.14)	0.033	170 ± 10	1.04(0.98,1.11)	0.185	167 ± 8	-	0.110
Weight(kg)		71 ± 15	1.02(0.98,1.06)	0.257	65 ± 16	0.99(0.95,1.03)	0.622	67 ± 12	-	0.228
Job (Unemployed)		14(47%)	0.46(0.16, 1.36)	0.163	17(57%)	0.69(0.23,2.05)	0.505	17(65%)	-	0.369
Completed Education Level										
High school		19(63%)	0.74(0.23, 2.36)	15(50%)	15(50%)	0.49(0.15,1.55)	18(69%)	-	-	
Bachelor’s degree		10(33%)	-	12(40%)	12(40%)	-	7(27%)	-	-	
Master's degree		1(3%)	0.70(0.04, 13.18)	3(10%)	3(10%)	1.75(0.15,20.23)	1(4%)	-	-	
Marital status (single)		21(70%)	1.87(0.59, 6.01)	0.290	14(47%)	0.58(0.20,1.71)	0.325	16(61%)	-	0.117
Smoking (yes)		8(27%)	2.92(0.68, 12.49)	0.148	8(27%)	2.79(0.65, 11.9)	0.166	3(11%)	-	0.282
Former substance abuse (Yes)		5(17%)	NC^d^	0.857	4(13%)	NC^d^	0.861	0(0%)	-	0.135
Family history of suicide (yes)		2(7%)	NC^d^	0.895	2(7%)	NC^d^	0.895	0(0%)	-	0.545
Self-harm history (yes)		12(40%)	NC^d^	0.767	7(23%)	NC^d^	0.785	0(0%)	-	0.002

^a^P-value(s) and Odds Ratio (s)Calculated based on univariate multinomial logistic regression model; ^b^Healthy controls group consider as a reference (Ref) in the multinomial logistic regression model;^**c**^P-value(s) calculated based on one-way analysis of variance /Kruskal–Wallis test or chi-square / Monte Carlo method. ^d^NC: not calculated because of sparse data some confidence intervals (95%CI) are wide

### CRP and BDNF

Mean level of BDNF was 17.74 ± 0.30 ng/ml in all subjects of the study. MD + SA, MD-SA, and healthy controls showed BDNF concentrations of 17.35 ± 2.43, 16.45 ± 3.15 and 19.65 ± 2.02 ng/ml, respectively. Analysis showed a significant difference between these groups in terms of BDNF level (p < 0.001). Post hoc pairwise analysis also revealed a significant difference between MD + SA group and healthy controls (p < 0.001) as well as MD-SA groups and healthy controls (p < 0.01) but no significant difference was found between MD + SA and MD-SA groups in terms of BDNF levels (p = 0.221).

Each unit increase in BDNF significantly decreased crude odds ofbelonging to the MDD + SA group by 32 percent (p-value = 0.002), and for being in the MDD group by 40percent compared to the control group. Moreover, it is 1.13percent more probable to be in MDD + SA than the MDD-SA group by each unit increase in BDNF level even though it is not statistically significant (OR:1.13; 95%CI (0.93. 1.37); P-value = 0.205).

Median (IQR) of CRP is 0.91(0.41–4.13) in MDD + SA, 1.11(0.39–2.95) in MDD-SA, and 0.87(0.39–2.95) in controls without any significant difference among them (p = 0.643).

Each unit CRP increase increases crude odds of being in MDD + SA for 1.04 percent, and MDD-SA by 1.03 percent without statistical significance. Moreover, it is 1.01 percent more probable to be in MDD + SA than MDD-SA group by each unit increase in CRP level even though it’s not statistically significant (OR:1.01; 95%CI (0.98. 1.04); P-value = 0.678) (Table 1).

To mitigate the risk of overfitting, a stepwise variable selection approach based on improvements in Akaike's Information Criterion (AIC) was employed to construct the final multiple multinomial logistic model. In this model, the healthy control group was designated as the reference category (Ref) (refer to Table 2). Based on final Model after considering covariates, each unit increase in BDNF decreases odds of being in MDD + SA and MDD-SA significantly compared to HC for 32% and 47% respectively. Each unit increase in CRP increases odds of belonging to MDD + SA for1.07%, and MDD-SA by 1.05percent without statistical significance.

**Table 2:** Final multinomial logistic regression model investigating predictors of MD + SA and MD−SAgroup

Variables	MDD + SA		MDD−SA	
	OR^+^ (95%CI)	P-value	OR^+^ (95%CI)	P-value
CRP (mg/L)	1.07 (1.0002, 1.15)	0.049	1.05 (0.98, 1.13)	0.164
BDNF (ng/ml)	0.68 (0.50, 0.93)	0.016	0.53 (0.39, 0.74)	< 0.001
Sex (female)	9.91 (1.77,55.5)	0.009	11.86 (1.89, 74.4)	0.008
Height(cm)	1.15 (1.08, 1.23)	< 0.001	1.17 (1.09, 1.26)	< 0.001
Weight(kg)	1.03 (0.96, 1.10)	0.384	0.93 (0.87, 1.001)	0.055
Marital status (single)	4.36 (0.59, 31.9)	0.147	0.15 (0.03, 0.93)	0.041
Smoking (yes)	0.97 (0.10,8.89)	0.975	5.40 (0.56, 52.35)	0.145

**Fig. 1:**
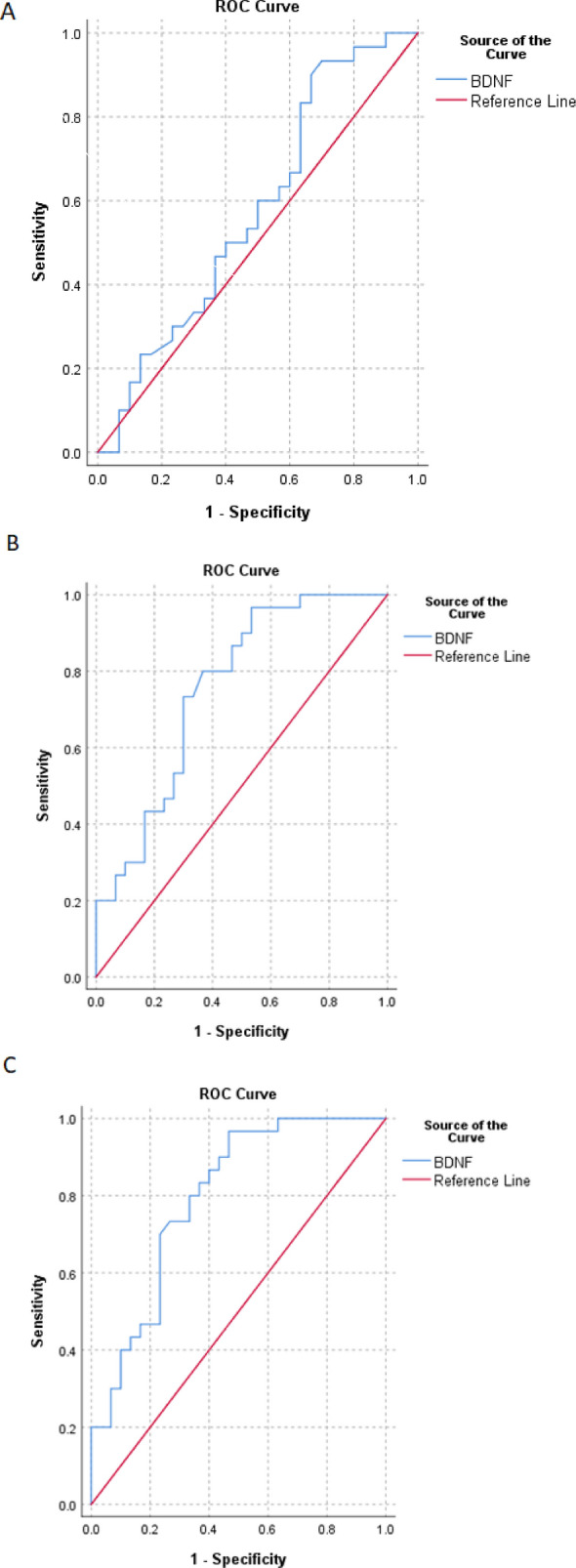
Receiver operating curve of MDD + SA group vs. MDD-SA group (**A**), MDD + SA group vs. control(**B**), and**.** MDD-SA group vs. control(**C**).BD, bipolar disorder; MDD, major depressive disorder; ROC, receiver operating curve

In our analysis, we utilized receiver operating characteristic (ROC) curves to assess the ability of predefined variables to effectively distinguish between MMD + SA, MDD-SA, and healthy control groups (as shown in Table 3).When examining the ROC curve for the discrimination of MMD + SA vs. MDD-SA based on BDNF levels, we observed an area under the curve (AUC) of 0.576 (95% confidence interval [CI] 0.429–0.722, p = 0.313). Furthermore, we determined that the optimal cutoff point for this discrimination was 16.66 (sensitivity: 60%, specificity: 50%) (refer to Fig. 1A). For the ROC curve analysis aimed at distinguishing MMD + SA from the healthy control group, we found an AUC of 0.754 (95% CI 0.632–0.877, p < 0.000) with a BDNF cutoff value of 18.43 (sensitivity: 73.3%, specificity: 70%) (see Fig. 1B). Lastly, when evaluating the ROC curve for differentiating MDD-SA from the healthy control group, we obtained an AUC of 0.796 (95% CI 0.683–0.909, p < 0.000). We determined that a BDNF cutoff point of 18.51, with a sensitivity of 73.3% and specificity of 70%, could effectively distinguish between these two groups (refer to Fig. 1C).

**Table 3:** ROC curve analysis for comparison of participants according to mental disease status

ROC analysis	MDD + SA group. MDD−SA group	MDD + SA group. control	MDD−SA group vs. control
AUC (95% CI)	(0.722–0.429) 0.576	(0.877–0.632) 0.754	(0.909–0.683) 0.796
*p* value	0.313	< 0.000	< 0.000
Cut-off	16.66	18.43	18.51
Sensitivity	60%	73.3%	73.3%
Specificity	50%	70%	70%

*ROC* receiver operating curve, *AUC* area under the curve, *MDD* major depressive disorder.

## Discussion

In this study, our primary objective was to investigate the association between two biological markers, BDNF and CRP, among patients suffering from MMD + SA and MDD-SA. Additionally, we aimed to assess the discriminatory capabilities of BDNF and CRP for distinguishing among individuals in the MMD + SA, MDD-SA, and healthy control groups.

We observed that there were no significant differences among the three groups in terms of demographic and baseline characteristics, except for a notable difference in the occurrence of self-harm. Specifically, self-harm was more prevalent in the MMD + SA group compared to the MDD-SA group. Interestingly, the control group did not report any history of self-harm.

Regarding the biological markers, we found that CRP levels did not exhibit significant variations between the groups. However, BDNF levels showed notable differences among the groups. BDNF concentrations in MDD patients, regardless of suicide attempts, were significantly lower than those in healthy controls. Importantly, BDNF demonstrated a significant ability to predict suicide attempts when compared to healthy controls, although it did not exhibit the same predictive power when compared to the MDD group.

When evaluating the performance of BDNF in discriminating between MMD + SA and MDD-SA groups against the healthy control group, ROC curve analysis indicated excellent discriminatory power. However, BDNF could not effectively differentiate between the MMD + SA and MDD-SA groups. In contrast, ROC curves for CRP did not demonstrate significant predictive power in our analysis.

Our findings regarding the association between BDNF and suicidal behavior, however, are inconclusive compared to the literature; In line with our findings, Eisen et al. in [16] used linear regression to show that no significant association was present between suicide attempt and BDNF level. Also, Eisen et al. in 2015 published a meta-analysis stating no significant correlation between BDNF levels and suicide attempts [17]. Moreira et al. [18] also indicated that BDNF levels were significantly lower in MDD patients with or without suicide attempts compared to healthy controls but no significant difference was found between depressed patients regardless of their suicide attempt history. Accordingly, it could be hypothesized that BDNF could be an indicator of depression not related to suicidal actions, and suicide attempts which not go on to complete suicide wouldnot have lower BDNF than depressed patients without suicide attempts.

Contrary to our results, Khan et al. [14] reported that even suicidal ideation in depressed patients is significantly correlated with lower BDNF levels compared to MDD-SA patients. Regression analysis also confirmed that no confounding factor influenced this significant relationship between BDNF and suicidal thoughts. Ai et al. [8] also showed that not only BDNF was significantly lower in MDD patients compared to controls, but also it was significantly lower in MDD + SA patients compared to MDD-SA patients. In that study, a significant correlation between BDNF concentration and suicidal thoughts or suicide attempts was found but BDNF level was not correlated with depression severity. Kudinova et al. [19] also emphasized the lower levels of plasma BDNF in cases with suicide attempt history compared to patients without any suicide attempt history. Both Ai et al. and Kudinova evaluated a history of suicide attempts not a recent history of suicide attempts as our participants. For instance, no participant in the Kudinova article reported a suicide attempt within the last year.

The discriminatory properties of BDNF in distinguishing between MDD + SA and MDD-SA groups have been explored in various studies, yielding different results. In our study, adjusted ROC curves based on regression models for BDNF exhibited excellent discriminatory ability for distinguishing the healthy control group from both the MMD + SA group (AUC = 0.754; 95% CI 0.877–0.632; sensitivity = 73.3%; specificity = 70%; cutoff = 18.43) and the MDD-SA group (AUC = 0.796; 95% CI 0.909–0.683; sensitivity = 73.3%; specificity = 70%; cutoff = 18.51). However, these adjusted ROC curves could not effectively differentiate between individuals with suicide attempts and those without (p = 0.313).In a study by Khan et al. [14], the most suitable cutoff point for BDNF levels between individuals with suicidal depression and those without was determined to be 444.58 pg/ml, with a sensitivity of 68.7% and specificity of 78.1%. However, no significant difference in BDNF levels was observed between the depressive control and normal control groups (p = 0.996).Lee et al. [20] reported that BDNF levels between MDD and normal control groups exhibited good discriminatory power using ROC curve analysis (AUC = 0.774; 95% CI 0.663 to 0.884; sensitivity = 78.3%; specificity = 81.3%; cutoff = 684.75). Moreover, suicidal MDD was significantly correlated with low BDNF levels, with an odds ratio of 33.123.

Regarding the discriminatory properties of BDNF in distinguishing between MDD and healthy control (HC) groups, Chiou and Huang [21] found that BDNF levels had poor discriminatory efficacy for depressed patients and HCs (AUC = 0.562, 95% CI 0.516 to 0.607). The optimal cutoff point for BDNF levels was 6.02 ng/ml, with a sensitivity of 72.8% and specificity of 41.8%. However, BDNF levels demonstrated moderate diagnostic power in the male subgroup (AUC = 0.652, sensitivity = 81.1%, and specificity = 48.5% at the BDNF level of 5.11 ng/ml) but not in the female subgroup (AUC = 0.536).In the study by Shahyad et al. [22], BDNF levels exhibited suitable discriminatory efficacy for distinguishing between depressed patients and HCs (AUC = 0.823; 95% CI 0.935–0.711; sensitivity = 80%; specificity = 83%; cutoff = 1206).

These findings suggest that the discriminatory properties of BDNF can vary across different studies and populations, highlighting the complexity of its role in distinguishing between various psychiatric conditions.

Altogether, our study in line with numerous previous reports [16–18], recognizes BDNF as a factor involved in the development of MDD without any association with suicide attempt while other studies, correlate BDNF levels with suicide attempt itself [8, 14, 19]. Our findings confirm the role of BDNF in the pathophysiology of depression but the correlation of BDNF and suicide is yet to be elucidated. It seems that these inconsistencies between studies originate from differences in study designs as depression severity; sample size, depression duration, and other parameters are probably different between studies and lead to these discrepancies. Importantly, ethnicity diversities should be considered and more evaluations ofdifferent regions are required for more reliable conclusions.

CRP levels were slightly different between the three groups of our study but these differences did not reach statistical significance level.CRP was not capable of predicting suicide or even depression noticeably. Our findings in this regard are not consistent with the majority of available studies. For instance, Goalkp et al. [23] have reported that CRP concentrations were significantly higher in the suicide group compared to controls. Kumar et al.[24] also confirmed this finding. Mohamed et al. [25] showed that even the presence of suicidal thoughts is significantly associated with CRP elevation. Courtlet et al.[26] also revealed that not only CRP is elevated in MDD + SA patiant but this relationship acts in a dose–response manner and increases in CRP levels are associated with a higher risk of suicide. Gibbs et al. [27] have also confirmed this linear relationship in which the level of CRP is positively correlated with lifelong suicide attempt frequency but it was not associated with suicidal thoughts.

As it can be concluded from the number of studies in recent years, abnormal inflammatory response has been a matter of focus in suicide research field. As a systemic inflammatory marker, association of CRP and MD + SA group can be somewhat result of a process in which increased CRP concentration can enhance blood–brain barrier permeability followed by entrance of CRP into the central nervous system and posing direct and indirect influences on CNS. In addition, prior research has shown that CRP can induce reactive activation of microglia and astrocytes and also rapid proliferation of glial cells which ultimately leads to neurons’ injury [28] These underlying processes may explain the association of CRP and suicide mentioned in other studies. Relationship of CRP and suicide was not observed in our study which could be due to small sample size and different study design. CRP can be confounded by various factors such as obesity, smoking, low vitamin D levels, low physical activity, inappropriate diet, allergy, stress, sleep disturbances and subclinical infections [29] which were not completely controlled in our study.

In the initial crude analysis, sex did not serve as a predictor. However, in the final model, being female was associated with higher odds of belonging to the MDD + SA and MD-SA group groups. This observation can be potentially explained by the higher prevalence of depression in women. Women tend to make suicide attempts more frequently than men, although they are more inclined towards attempting suicide rather than completing it. In contrast, men are more likely to carry out suicide and often employ more violent methods. Consequently, women can be characterized as the “attempters” and “survivors” of suicide attempts [30].

In this study, an individual's height was found to elevate the odds of being part of both the MDD and MDD + SA groups. When it comes to the connections between depression and height, the existing body of research presents a limited and conflicting set of findings. Some researchers propose that height, particularly shorter stature, may act as a causal risk factor for depression [31], while others hold a differing viewpoint [32, 33]. AlsoThere was inverse association between height and suicide riskin some studies [34]. Height serves as an indicator of health status, susceptibility to specific illnesses, and overall quality of life. The prevalent inclination to link taller stature with notions of physical attractiveness, authority, leadership, enhanced cognitive abilities, and achievements in both social and professional domains fosters the belief that, in the context of human growth, akin to economic growth, larger is deemed superior [35]. Individuals of shorter stature are more prone to occupy lower social class positions in adulthood, irrespective of their childhood social class. This lower social class status is linked to an increased risk of suicide [36]. These insights could provide a rationale for the observed connection between height and suicide attempts or MDD as highlighted in certain research studies.Regarding the inconsistency in research findings, it is plausible to suggest that there might be undisclosed moderating variables influencing the relationship between height and depression.

In the initial crude analysis, marital status (being single) did not emerge as a predictive factor. However, in the final model, being single was associated with a decreased likelihood of being in the MDD group. These outcomes do not align with previous findings in this context [37, 38]. In considering a potential explanation for this discovery, it’s worth noting that various variables such as the quality of relationships, cultural factors, and socioeconomic status could potentially act as moderators in the relationship between MDD and marital status.

In this study, a noteworthy difference was observed in the history of self-harm among the various groups.Importantly, none of the participants in the control group reported any history of self-harm. while self-harm is not limited exclusively to individuals with depression, it is notably more prevalent among individuals who experience depression or those who have attempted suicide. [39]; When considering potential explanations for this finding, it’s conceivable that the choice of sample source, whether drawn from the general population or admitted psychiatric patients, could have influenced the study’s outcomes. Additionally, the unique religious beliefs and cultural context in Iran may play a role. In Iranian society, self-harm is both forbidden and stigmatized, which may lead to underreporting of such behaviors among the general population [40].

To our knowledge, this study is the first to simultaneously assess BDNF and hs-CRP levels in Iranian patients with MDD + SA and MD-SA group, all of whom were drug-free. We excluded individuals who had previously used mood stabilizers or antidepressants due to their potential impact on BDNF and hs-CRP levels. However, there are limitations: our study design couldn’t establish causal relationships between the parameters, and we didn’t consider factors like sleep problems, socio-demographics (rural vs. urban living), seasonality, or illness duration and severity, all known to affect BDNF expression [22].Due to the illegality of alcohol consumption in Iran and potential inaccuracies in participants’ self-reporting, alcohol usage was not considered in our assessment. The patients were not monitored for the occurrence of other mental episodes, primarily because suicide patients, who were hospitalized, were less inclined to participate in the research. Motivating participation in questionnaire completion posed a challenge, and we took care not to seek consent from heavily medicated or emotionally distressed patients incapable of providing informed consent. In such instances, our study personnel contacted the hospital later when the patient was stable. Furthermore, we did not analyze post-mortem blood samples from individuals who completed suicide due to hospital regulations. Consequently, the generalization of our findings to all suicide cases should be done cautiously. Lastly, we measured serum BDNF levels, recognizing that BDNF can traverse the blood–brain barrier, and there is a suggested positive correlation between serum and cortical BDNF levels.

## Conclusions

In our study, the concentration of BDNF differed significantly between depressed patients with/without suicide attempts and healthy controls which show the association of BDNF with depression development itself and not suicide attempt. We could not find any association between CRP levels and suicide attempts but still larger cohorts are needed for a definite conclusion.

## Data Availability

The data generated and/or analyzed during the current study are available from the corresponding author or reasonable request.

## Footnotes

1 SCID-5.
